# TCM nonpharmacological interventions for chronic obstructive pulmonary disease (COPD): Protocol for a systematic review and network meta-analysis

**DOI:** 10.1097/MD.0000000000015979

**Published:** 2019-06-07

**Authors:** Furong Zhang, Xixi Chen, Xiaoyan Wu, Xicen Liu, Mingsheng Sun, Xiaoyu Shen, Juan Li, Hui Zheng, Rongjiang Jin

**Affiliations:** a College of Health Preservation and Rehabilitation; b College of Acupuncture and Tuina, Chengdu University of Traditional Chinese Medicine; c Nuclear Industry 416 Hospital, Chengdu city, Sichuan province, China.

**Keywords:** chronic obstructive pulmonary disease, network meta-analysis, study protocol, TCM nonpharmacological interventions

## Abstract

**Background:**

Traditional Chinese medicine (TCM) nonpharmacological interventions are gaining an increasing popularity for chronic pulmonary obstructive disease (COPD) treatment and rehabilitation, yet their comparative effectiveness and safety remains unclear. Therefore, this study will aim to compare their effectiveness and safety for COPD by implementing a network-meta analysis.

**Methods:**

Electronic databases including MEDLINE via Ovid, and EMBASE via Ovid, the Cochrane Central Register of Controlled Trials (CENTRAL), China National Knowledge Infrastructure (CNKI) and Chinese BioMedical Literature Database (CBM) will be searched for related randomized controlled trials (RCTs) from inceptions to the search date without language restrictions. RCTs that compare TCM nonpharmacological interventions with placebo or conventional treatments will be included. The primary outcome will be lung function measures, dyspnea level and COPD-specified life quality and secondary ones will include functional exercise capacity, and general health-related life quality. Both classical meta-analysis and network meta-analysis will be implemented to investigate direct and indirect evidences on this topic. Continuous data will be expressed as standard mean differences (SMDs) and categorical data as risk ratios (RRs) with 95% confidence intervals. The evidence transitivity and consistency within network will be evaluated with Cochrane Q statistic and net-heat plot.

**Results:**

The study results will be disseminated through a peer-reviewed journal publication or conference presentation.

**Conclusions:**

The review findings will provide a ranking evidence of current TCM nonpharmacological interventions and help to inform clinical practitioners, COPD patients, and policy-makers in the decision-making.

**Trial registration number:**

PROSPERO CRD42019126554

## 1 Introduction

Chronic obstructive pulmonary disease (COPD) is a common chronic respiratory condition caused by significant exposure to noxious discharges and host factors, and it brings about obvious persistent respiratory symptoms and high incidence of morbidity and mortality.^[1–4]^ It is now ranking the fourth leading cause of death worldwide with an estimation of 65 million COPD sufferers according to WHO, imposing heavy health and socio-economic burdens globally.^[5,6]^ According to the Global Initiative for Chronic Obstructive Lung Disease(GOLD) standard, COPD is classified into four stages based on the severity of airflow limitation measured by spirometry results; Levels from Gold 1 to Gold 4 are corresponding to degrees of mild, moderate, severe and very sever respectively. However, evidence shows that the correlation between FEV1, clinical symptoms and health status impairment is weak, therefore, symptomatic and risk factor assessments are also of great importance.^[7]^ Currently, apart from reducing exposure to risk factors like smoking, air pollution and occupational threats, mainstream guidelines recommend pharmacological therapies as main interventions for stable COPD management, especially for symptom alleviation, risk and exacerbation palliation as well as health status and exercise tolerance improvement. Single or combined utilization of long-acting bronchodilator or anti-inflammatory agents are individually tailored according to patients’ situation.^[1]^ Though with standard and regular western medicine treatment, the effects of proportional COPD patients turned out to be not that satisfactory, some researches even suggests that the medication safety of long-acting β_2_-agonists (LABAs) remains indefinite among victims to COPD and might increase death risk in patients with asthma.^[8–10]^ Therefore, it urges other potential strategies to help slow down the development and deterioration of this disease.

Under the circumstance, complementary and alternative medicine (CAM) has been gaining more attention in the COPD treatment worldwide. Traditional Chinese medicine (TCM), as a main component of CAM based on current knowledge, has been widely applied in management of chronic conditions including COPD in its birth place, China. Recently, an increasing amount of evidenced -based medicine (EBM) evidences have revealed that TCM therapies including acupuncture, traditional Chinese exercises like *Tai Chi*, *Baduanjin*, and Chinese herbal medicine, have potentially positive effects in COPD management,^[11–16]^ yet no comparative effectiveness investigation was done, which may exert some influence on clinical decision-making and health-economic policies-implementing. Due to the complex components and working mechanisms of orally-administrational herbs and to avoid extra pharmacological intake load which may bring digestive problems to patients, we only aim to examine comparative effectiveness of TCM nonpharmacological interventions in COPD by conducting a systematic review and network meta-analysis.

## 2 Methods

### 2.1 Protocol register

This protocol has been prepared under the guidance of the Preferred Reporting Items for Systematic Reviews and Meta-Analyses Protocols guidelines,^[17]^ and it has been registered on PROSPERO platform (https://www.crd.york.ac.uk/PROSPERO/) with an assigned registration number CRD42019126554.

### 2.2 Ethics

For all eligible studies were approved by local institutional review boards and ethical committees, and participants included were required to complete written informed consents, this study requires no further ethical approval.

### 2.3 Information sources and search strategy

Electronic databases including MEDLINE via PubMed, and EMBASE via Ovid, the Cochrane Central Register of Controlled Trials (CENTRAL), China National Knowledge Infrastructure (CNKI) and Wanfang Database will be searched for related randomized controlled trials (RCTs) from inceptions to 3 January 2019 without language restrictions. A systematic search will be carried out by using combination of medical subject headings (MeSH) and keywords; A preliminary search strategy is provided in Table 1, which will be adapted according to syntax-related requirements of the electronic databases. The ongoing RCTS will be searched in clinical trials registries, such as World Health Organization International Clinical Trials Registry Platform (WHO ICTRP) and clinicaltrials.gov and we will contact study authors to identify additional studies if necessary. The reference about to relevant systematic reviews will be screened, and a manual search of respiratory journals and meeting abstracts will be conducted to check for other possibly relevant articles.

**Table 1:**
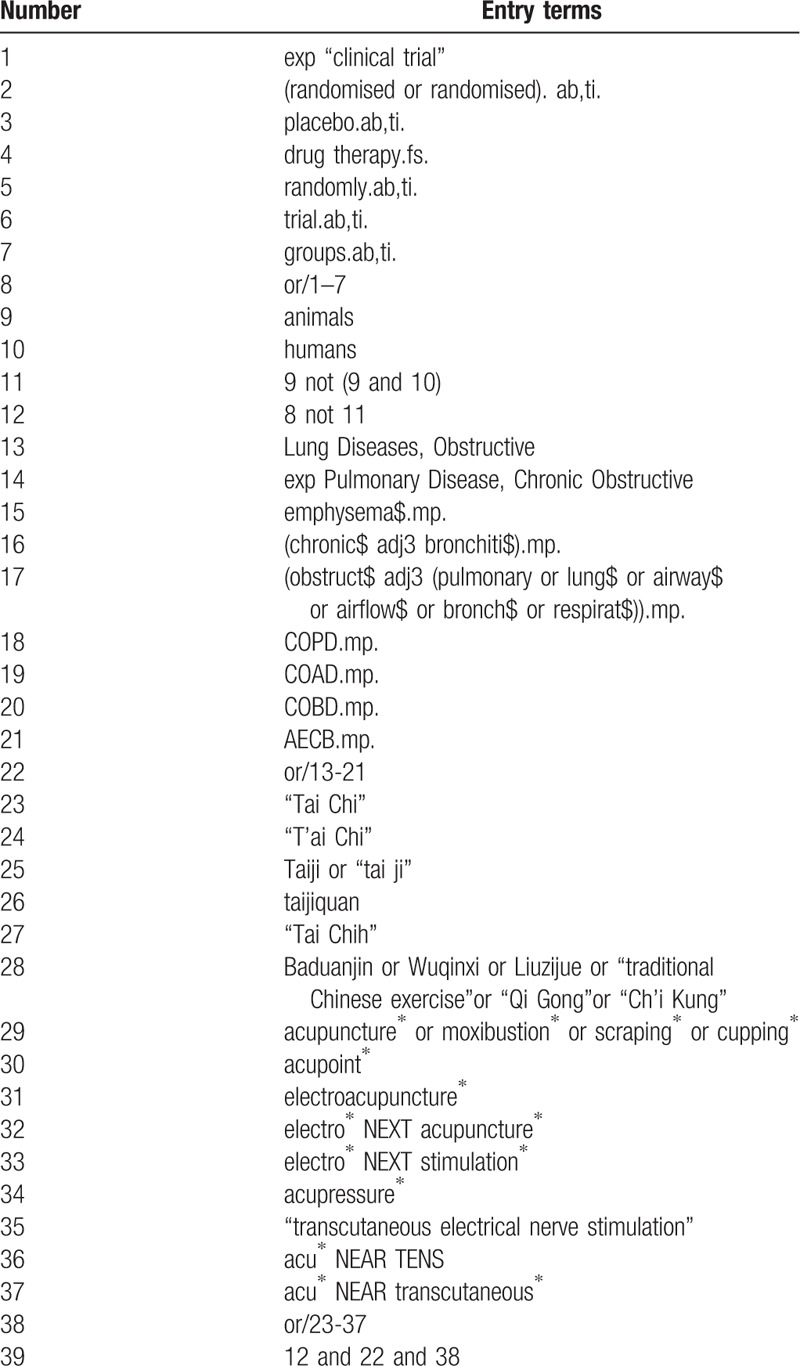
Search strategy draft.

### 2.4 Eligibility criteria

The PICOS (participant, intervention, comparison, and study design) principle has been applied in the study design.

#### 2.4.1 Study design

We will only include parallel designed randomized controlled trials (RCTs). No language restrictions will be applied. Studies with non-RCT design, quasi-experiment design, unobtainable data, duplicate publications, animal experiments and reviews or case reports will be excluded.^[18]^

#### 2.4.2 Participants

Adult patients (≥18 years old) clinically diagnosed with COPD (post-bronchodilator FEV1/FVC<0.7) in stable stage (mild to moderate, GOLD1 to GOLD2) will be included. Being stable means that patients are in a relatively mild or moderate state according to GOLD standard, to put in another way, they are on regular respiratory medications with no exacerbation or hospital admission within the previous month, although oxygen supplementation during training could have been used.^[19]^ Besides, patients with severe comorbidities like cerebral cardiovascular disease, skeletal muscle dysfunction, osteoporosis and so forth will be excluded if COPD data cannot be accessed specifically.

#### 2.4.3 Interventions and comparators

TCM non-pharmacological interventions for COPD will be included, including acupuncture, moxibustion, cupping, *tuina*, scraping, traditional Chinese exercise like *Taichi*, *Baduanjin*, *Wuqinxi* and so forth. Eligible treatments can be used as monotherapy or combined treatments, which should be given consecutively for at least for a month. Studies of pharmacological-related treatment (e.g., acupoint application therapy, medicated bath, medicated fumigation) will be excluded. Comparators will include placebo, pulmonary rehabilitation, and other positive interventions. Studies comparing the same kind of TCM nonpharmacological interventions, but with different sessions, acupoints selections will be taken as the identical node in network analysis.^[20]^

#### 2.4.4 Outcomes

##### 2.4.4.1 Primary outcomes

1. The lung function including FEV1, FVC and FEV1/FVC.
2. Dyspnea level assessed by validated tools like Modified Borg Scale, Modified Medical Research Council Dyspnea Scale.
3. Change in COPD-specific life quality measured by tools like the St Georges Respiratory Questionnaire (SGRQ) or the COPD Assessment Test (CAT).^[21]^

##### 2.4.4.2 Secondary outcomes

Secondary outcomes will include functional exercise capacity such as 6-minute walk test (6MWT), endurance cycle test; Muscle strength like maximal voluntary contractions (MVCs); common health-related life quality measured by validated tools like 36-Item Short Form Health Survey; Prognosis indexes including exacerbations, hospital admissions and death. Besides, safety measurement, adverse event and costs will also be considered.

### 2.5 Selection of studies and data extraction process

**Figure 1:**
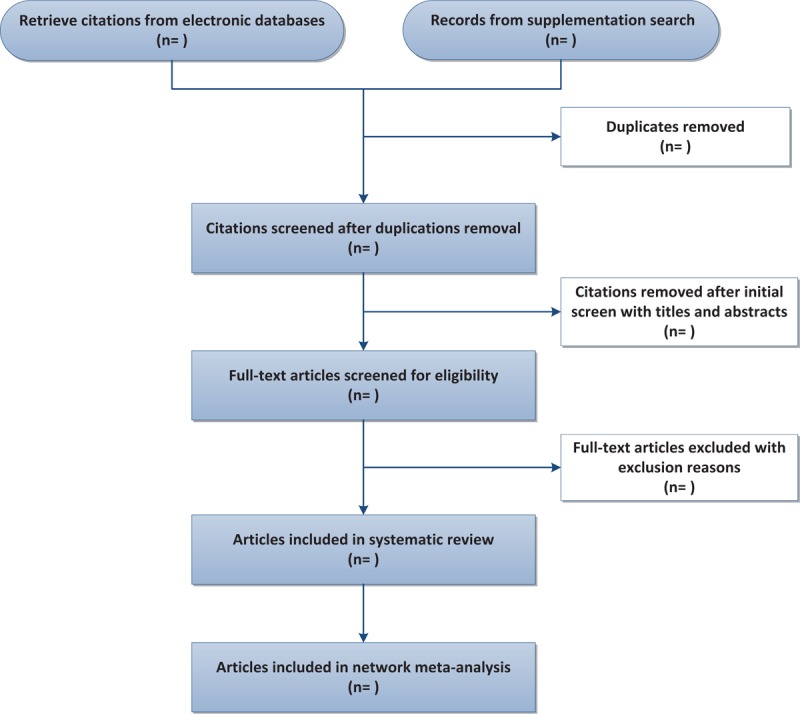
This flow diagram is based on PRISMA framework, which shows the whole process of literature search in this study, including records retrieving, screening, inclusion, exclusion with reasons and articles included in final qualitative and quantitative analysis.

Study selection and data extraction process will be independently conducted and cross-checked by 2 authors, respectively (FRZ and XXC). All retrieved literature citations will be imported and managed in Endnote X7 (Bld 7072). A standard pre-designed form will be used for date extraction, with items including first author, country, year of publication, number of centers and the participants, study design, number of groups, allocation ratio, interventions, comparisons, outcomes (primary and secondary outcomes) and conclusions. Discrepancies between the 2 reviewers will be settled by discussion or the introduction of a third reviewer (RJJ). The flow chart based on PRISMA^[22]^ is displayed in Figure 1.

### 2.6 Risk of bias assessment

The risk of bias of each study included will be evaluated independently by 2 investigators (XYW and XCL) according to the Cochrane ‘Risk of Bias’ assessment tool, which focuses on 6 domains including sequence generation, allocation concealment, blinding, incomplete data, selective reporting and other bias. Disagreements between the 2 authors will be resolved by discussion. If the disagreement persists, a third reviewer (JRJ) will be consulted to reach consensus.

### 2.7 Data synthesis

According to the PICOS principle, main characteristics of included study will be qualitatively summarized, such as study type, participants, interventions and outcomes. The risk of bias associated with missing values will be fully considered, and the missing values that cannot be obtained from the RCT authors will be settled according to the Cochrane handbook.

Continuous and categorical data will be expressed as standard mean differences (SMDs) and risk ratios (RRs) respectively, with 95% confidence intervals (95% CIs). Random effect model will be selected in data pooling. Conventional pair-wise meta-analysis will be performed for direct comparisons with 95%CIs.

A network meta-analysis based on frequent framework will be conducted by using the *mvmeta* and *network* packages in Stata software (Stata V.14.0, StataCorp). A network diagram will be made to visualize the numbers and interrelations of interventions included. The interventions results will be ranked based on their surface under the cumulative ranking curve (SUCRA), by evaluating the certainty extent of intervention superiority without resampling.^[23,24]^

We will examine the consistency of network meta-analysis by comparing the direct and indirect evidences with *Z* test. The Cochrane *Q* statistic will be employed to assess heterogeneity.^[25]^ If with homogeneity, a fixed-effect model will be adopted; If with obvious heterogeneity, a random-effect model will be applied and a further meta-regression analysis will be performed to determine their potential sources. A contribution matrix will be performed to demonstrate percentage contribution of each direct comparison to the whole evidence body. Besides, sensitivity analysis will also be implemented by excluding studies with poor-quality or high risk of bias and PP set based RCTs. Publication bias will be examined with a funnel plot if with more than 10 studies included. If it needs to be quantified, Begg method or Egger method will be carried out.

### 2.8 GRADE quality assessment

GRADE framework^[26]^ will be utilized to assess the quality of evidence concerning main outcomes and recommendation strength of related interventions by two independent authors (FRZ and XXC). Confidence in each network estimates will be degraded from High to Moderate, Low or Very Low, according to assessment results of the five domains including study limitations, inconsistency, indirectness, imprecision and publication bias.

## 3 Discussion

With an increasing amount of publications on nonpharmacological interventions for patients with COPD in recent years, we would like to figure out which one has the relatively optimal effect and safety among those interventions. Given that systematic reviews with good quality can help provide best evidence in clinical practice, and a network meta-analysis can offer a ranking result based on comparative effectiveness, safety and costs. Therefore, we conceive and design this study protocol. We also hope that the study result will give some new insight into this field, which, to some extent, can help clinical physicians, patients and their family, and policy-makers to make better choice.

## Author contributions

**Conceptualization:** Furong Zhang, Xixi Chen, Rongjiang Jin.

**Data curation:** Xiaoyan Wu, Xicen Liu.

**Formal analysis:** Furong Zhang, Xixi Chen, Hui Zheng.

**Investigation:** Mingsheng Sun, Xiaoyu Shen.

**Methodology:** Xiaoyan Wu, Juan Li, Hui Zheng.

**Resources:** Mingsheng Sun, Rongjiang Jin.

**Software:** Xiaoyu Shen, Juan Li.

**Supervision:** Rongjiang Jin.

**Writing – original draft:** Furong Zhang, Xixi Chen, Xiaoyan Wu, Xicen Liu.

**Writing – review & editing:** Hui Zheng, Rongjiang Jin.

Furong Zhang orcid: 0000-0002-4671-2295.
